# PTEN activity could be a predictive marker of trastuzumab efficacy in the treatment of ErbB2-overexpressing breast cancer

**DOI:** 10.1038/sj.bjc.6602926

**Published:** 2006-01-10

**Authors:** T Fujita, H Doihara, K Kawasaki, D Takabatake, H Takahashi, K Washio, K Tsukuda, Y Ogasawara, N Shimizu

**Affiliations:** 1 Department of Cancer and Thoracic Surgery, Okayama University Graduate School of Medicine and Dentistry, 2-5-1 Shikata-cho, 700-8558 Okayama, Japan

**Keywords:** breast cancer, erbB2, trastuzumab, PTEN, Akt

## Abstract

Trastuzumab is the only HER2/neu-directed therapy to have received Food and Drug Administration approval for the treatment of patients with metastatic breast cancer. The efficacy of trastuzumab depends on the *HER2/neu* status of the tumour and the patient's prior treatment, but even when patients are selected on the basis of *HER2/neu* gene amplification, the single-agent response rate ranges from 12 to 30% and few patients respond to trastuzumab monotherapy. Here, we propose PTEN as a predictive biomarker for trastuzumab efficacy. Human breast cancer SKBR3 and drug-resistant SKBR3/R cells were investigated. We also examined clinical samples from patients who had been treated with trastuzumab and analysed the relationship between trastuzumab efficacy and PTEN level. The PI3K/Akt signalling pathway was observed to be highly active in the drug-resistant cells, and their level of PTEN was low. Delivery of antisense *PTEN* duplex siRNA significantly decreased the trastuzumab chemosensitivity of parental SKBR3 cells, and marked activation of Akt signalling pathway was also recognised. Moreover, immunohistochemical investigation revealed that trastuzumab treatment was remarkably successful in cells with elevated PTEN expression. Along with the immune-system-associated cytotoxic mechanism, several mechanisms have been proposed for the effect of trastuzumab. PTEN activity might play an important and major role in its HER2/PI3K/Akt-mediated antitumour effect, and could be a useful biomarker for predicting the efficacy of trastuzumab in the treatment of breast cancer.

*PTEN* (phosphatase and tensin homologue deleted on chromosome 10, also known as *MMAC* and *TEP1*) is a recently discovered tumour-suppressor gene located on chromosome 10q23.3. *PTEN* mutations have been implicated in variety of human cancers including endometrial cancer (30–50%) (Risinger *et al*, 1997; Tashiro *et al*, 1997), high-grade glioma (60–80%) (Louis and Gusella, 1995) and prostate cancer (29%) (Cairns *et al*, 1997), and homozygous deletion of the *PTEN* gene causes embryonic lethality (Podsypanina *et al*, 1999). PTEN also antagonises phosphatidylinositol 3 kinase (PI3K) function and negatively regulates Akt activity. Restoration of PTEN expression in PTEN-null cells inhibits Akt activity and tumour formation (Li and Sun, 1998; Lu *et al*, 1999).

The *HER2/neu* oncogene, the second member of the epidermal growth factor receptor family, encodes a transmembrane tyrosine-kinase receptor. Overexpression of HER2/neu, which is seen in approximately 30% of breast cancers, is associated with poor overall survival (Yu and Hung, 2000) and in particular with increased metastatic potential and resistance to chemotherapeutic agents. Several reports have described the significance of PI3K and the Akt pathway in HER2/neu signalling. PI3K and Akt have been shown to play important roles in proliferation and cell survival and induce the expression of many cytokines (Downward, 1998).

Recent studies have demonstrated that resistance to trastuzumab treatment depends on the level of PTEN present (Crowder *et al*, 2004; Nagata *et al*, 2004; Pandolfi, 2004), with Nagata *et al* (2004) demonstrating that PTEN deficiency confers trastuzumab resistance in HER2/neu-overexpressing breast cancer cells.

Here we present the use of PTEN for predicting the efficacy of trastuzumab in drug-resistant and parental HER2/neu-overexpressing breast cancer cells. We also investigate the expression of PTEN in a clinical setting and discuss its role.

## MATERIALS AND METHODS

### Cell culture and reagents

Human breast cancer SKBR3 cells were obtained from the American Type Culture Collection (Manassas, VA, USA) and maintained in Dulbecco's modified Eagle's medium supplemented with fetal bovine serum (10% v v^−1^), penicillin (100 IU ml^−1^) and streptomycin (100 *μ*g/ ml^−1^) incubated in 5% CO_2_. Trastuzumab resistance was developed by continuous exposure to trastuzumab (4 *μ*g ml^−1^ for SKBR3/4R and 8 *μ*g ml^−1^ for SKBR3/8R) for 4 months, during which time the medium was replaced every 5 days and cells were passaged when 50% confluence was reached. Trastuzumab resistance was confirmed by dose–response studies as described below.

Trastuzumab was purchased from Chugai Seiyaku (Tokyo, Japan).

### Western blot analysis

Cells (5–10 × 10^6^) were washed twice with phosphate-buffered saline (PBS), then lysed with a hypotonic HEPES buffer (250 *μ*l, 10 mM, pH 7.6) containing KCl (50 mM), phenylmethane sulphonyl fluoride (0.1 mM), aprotinin (0.5 *μ*l ml^−1^) and sodium orthovanadate (0.1 mM). After centrifugation, supernatants were adjusted to contain equal concentrations of protein, diluted with one volume of 5 × SDS sample buffer and heated for 5 min at 95°C. Samples (30 *μ*g protein) were run on a 4–20% SDS–PAGE gel and electroblotted onto polyvinylidene fluoride membrane. Blots were blocked overnight in blocking solution (5% nonfat milk powder, 0.1% Tween 20 in PBS) at 4°C. Afterwards, the blots were exposed to antibodies against HER2, PI3K, Akt, PTEN (Santa Cruz, CA, USA), p-PI3K or p-Akt (Cell Signalling) at 1000-fold dilution in blocking solution (1 h, room temperature). After extensive washing with blocking solution, blots were exposed to the appropriate secondary antibody at 10 000-fold dilution in blocking solution. After extensive washing, blots were examined using an enhanced chemiluminescence detection method (ECL kit, Amersham Pharmacia Biotech, Chandler, AZ, USA).

### Determination of cytotoxicity by MTT (3-[4,5-dimethylthyazol-2-yl]-2,5-diphenyl tetrazolium bromide) assay

Cells were seeded at a concentration of 3 × 10^3^ cells per well in flat-bottomed 96-well microplates. After 24 h, the cells were cultivated with trastuzumab for 120 h or paclitaxel for 12 h, or left untreated, then their viability was determined using Promega's CellTiter 96® Aqueous One Solution Cell Proliferation Assay (Promega Corp., Madison, WI, USA).

### Delivery of *PTEN* duplex siRNA *in vitro*

Duplex siRNA against *PTEN* (AF143314 4688 E06: 5′-AUGCCAACAACAAGCUUCUUACAAUGCC-3′) and control duplex siRNA (AF143314 4688 E07: 5′-AUGUACCAACCGAAUCUUACAUGCC-3′) (Life Technologies, Rockville, MD, USA) were delivered in drug-sensitive parental SKBR3 cells. Cells were plated in 100 mm dishes at 30% confluence and transfected with duplex siRNA (25 nM) using Oligofectamine (Life Technologies, Rockville, MD, USA) 48 h postplating. Cells were replated for individual assay 96 h postplating. PTEN expression was determined 120 h postplating and growth inhibition assay was performed after 72 h postplating followed by incubation with chemotherapeutic agents.

### Evaluation of HER2/neu status

HER2/neu status was determined based on gene amplification and/or immunohistochemical evaluation. *HER2/neu* gene amplification of the patients' samples was determined by fluorescence *in situ* hybridisation (FISH) using the PathVysion FISH assay (Vysis, IL, USA). Immunohistochemically, HER2/neu status was determined by Herceptest (DAKO, Tokyo, Japan).

### Immunohistochemical investigation of PTEN

Clinical samples were used for immunohistochemical investigation with PTEN. Each sample was obtained from a surgical specimen of a patient who had received trastuzumab treatment in combination with paclitaxel in our department between 2001 and October 2005. For immunohistochemistry, paraffin sections were stained after microwave treatment by an intact method. Antibody against PTEN (Santa Cruz, CA, USA) was exposed overnight at 4°C followed by treatment with the LSAB2 kit (DAKO Carpinteria, CA, USA) according to the manufacturer's instructions. The PTEN expression level was scored semiquantitatively based on staining intensity and distribution using the immunoreactive score (IRS) as described elsewhere (Friedrichs *et al*, 1993; Chui *et al*, 1996); briefly, IRS=SI (staining intensity) × PP (percentage of positive cells). Staining intensity is assigned as 0, negative; 1, weak; 2, moderate; 3, strong. Percentage of positive cells is defined as 0, <1%; 1, 1–10%; 2, 11–50%; 3, 51–80%; and 4, >80% positive cells. In all, 10 visual fields from different areas of each tumor were used for the IRS evaluation. Negative control slides without primary antibody were included for each staining. Normal breast epithelium or vascular endothelium known to express normal PTEN was used as positive control. Based on the IRS score, staining intensity was graded − (IRS 0–3), 1+ (IRS 4–6), 2+ (IRS 7-9) or 3+ (IRS 10–12).

### Statistical analysis

Levels of statistical significance were evaluated using data from at least three independent experiments by using the two-tailed Student's *t* test, Fisher's test and ANOVA. *P*<0.05 was considered to be statistically significant. All data were analysed using StatView for Windows (SAS Institute Inc., Cary, NC, USA).

## RESULTS

### Growth inhibition of trastuzumab and paclitaxel in parental and drug-resistant cells

**Figure 1:**
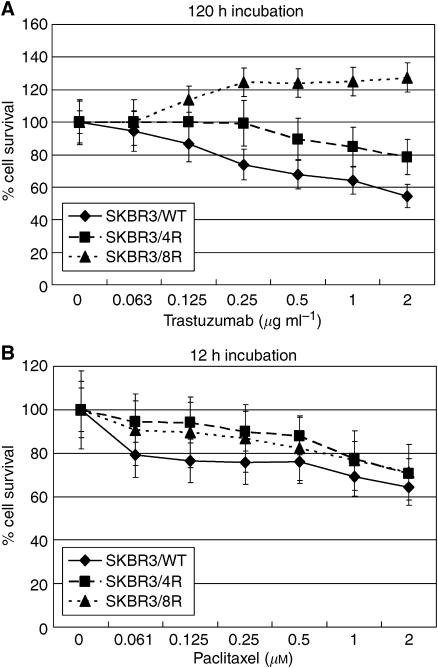
(**A**) Growth inhibition assays revealed that chemosensitivity to trastuzumab was decreased in drug-resistant SKBR3/4R and /8R cells (120 h incubation with trastuzumab after 24 h incubation with medium). (**B**) Chemosensitivity of paclitaxel was not significantly different in drug-resistant and parental SKBR3 cells (12 h incubation with paclitaxel after 24 h incubation with medium).

**Figure 2:**
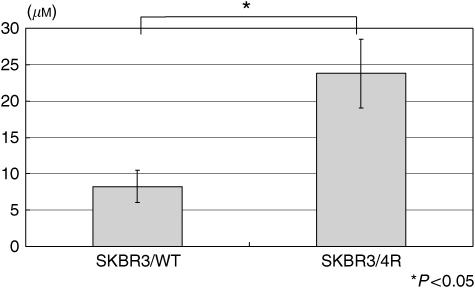
The IC_50_ value of trastuzumab in drug-resistant SKBR3/4R cells was statistically significantly higher than in parental cells.

The HER2/neu-overexpressing SKBR3 cells were treated with serial dilutions of trastuzumab and paclitaxel. The cells' survival at various drug concentrations are shown in Figure 1. In the drug-resistant cells, mean cell viability was 79.6% (SKBR3/4R) and 128.6% (SKBR3/8R) at 2 *μ*g ml^−1^ trastuzumab, which is markedly higher than the wild-type (SKBR3/WT) parental cells. Mean cell viabilities (IC_50_) in parental and resistant SKBR3/4R cells were 8.25 and 23.79 *μ*M trastuzumab, respectively; the difference was statistically significant (Figure 2). However, the response of drug-resistant and parental cells to paclitaxel treatment did not differ significantly.

### Western blotting analysis of HER2/neu expression and downstream signal proteins

**Figure 3:**
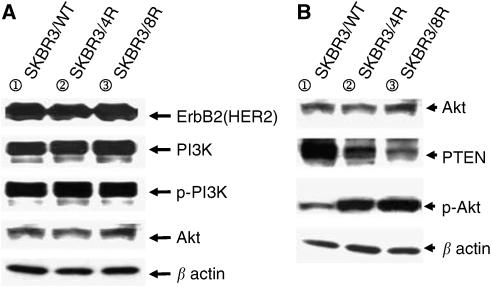
(**A**) Western blotting analysis revealed that the level of expression of the ErbB2/PI3K/Akt signal transduction pathway is the same in trastuzumab-resistant and parental cells. (**B**) Phosphorylated Akt activity was increased in drug-resistant SKBR3/R cells; PTEN expression was decreased in these cells.

Western blot was performed to detect expression of the downstream cascade pathway of HER2/neu2 in both resistant and parental cells. Expression of HER2/neu, PI3K, phosphorylated PI3K and nonphosphorylated Akt were the same in both cell types (Figure 3a). However, expression of phosphorylated Akt was significantly higher in the drug-resistant cell lines. Conversely, PTEN expression was decreased in the drug-resistant cells and this downregulation was more pronounced in the more resistant cells. Owing to the downregulation of PTEN, expression of phosphorylated Akt was increased and signal transduction of the Akt-mediated pathway was activated (Figure 3b).

### PTEN deficiency and trastuzumab sensitivity in parental cells

**Figure 4:**
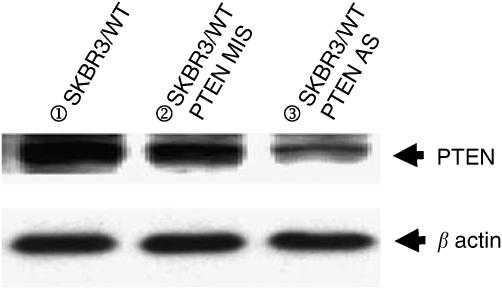
Western blotting analysis of antisense-delivered SKBR3/WT cells showed that expression of PTEN was inhibited by duplex antisense-siRNA delivery.

**Figure 5:**
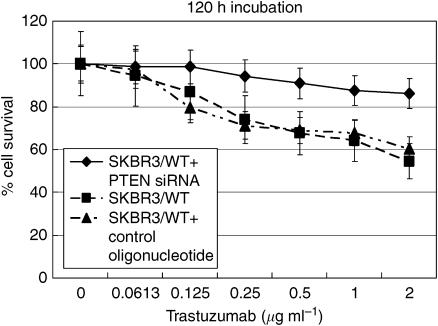
The growth inhibition curve of duplex antisense-siRNA-delivered SKBR3/WT cells shows decreased trastuzumab sensitivity (120 h incubation of trastuzumab 72 h postplating).

To elucidate the effect of PTEN on trastuzumab's efficacy, SKBR3/WT, 4R and 8R cells were transfected with *PTEN* antisense oligonucleotides, which effectively reduced endogenous PTEN expression compared with the cells transfected with control mismatched (MIS) oligonucleotides (Figure 4). To investigate whether PTEN activation contributes to trastuzumab's antiproliferation function, we compared cell growth between MIS control and *PTEN* antisense-delivered SKBR/WT cells after trastuzumab treatment. *PTEN* antisense-delivered SKBR3/WT cells, which had reduced PTEN expression, showed significantly less growth inhibition with trastuzumab than MIS control-delivered cells with a normal level of PTEN expression (Figure 5).

### Western blotting analysis of HER2/neu expression and downstream signal proteins in *PTEN*-deficient cells

**Figure 6:**
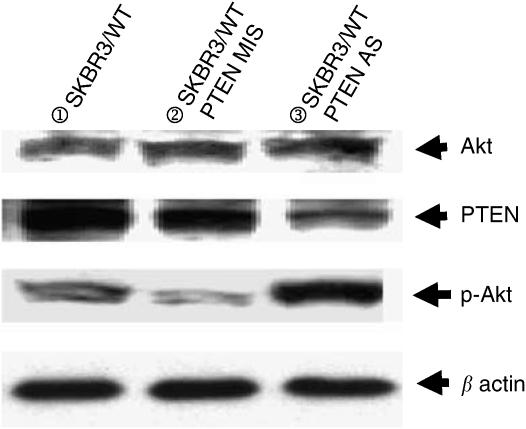
Western blotting analysis of antisense-delivered SKBR3/WT cells showed that expression of phosphorylated Akt was increased. This suggests that Akt activity is partly due to the level of PTEN in ErbB2-overexpressing SKBR3 cells.

Western blot was used to detect the expression of the downstream cascade pathway of HER2/neu in *PTEN* antisense-delivered cells. Expression of nonphosphorylated Akt was the same in all cells, but expression of phosphorylated Akt was significantly higher in antisense-delivered SKBR3/WT cells than in drug-resistant cells (Figure 6).

### Growth inhibition effect of paclitaxel in *PTEN*-deficient cells

**Figure 7:**
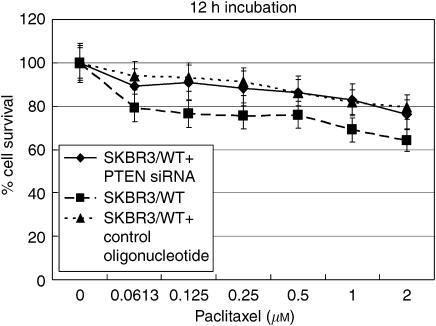
The growth inhibition rate of paclitaxel in antisense-delivered SKBR3/WT cells. No significant difference was observed for the growth inhibition effect of paclitaxel between antisense- and control oligonucleotide-delivered cells (12 h incubation of paclitaxel 72 h postplating).

The growth-inhibition effect of paclitaxel was analysed in *PTEN*-deficient cells. No significant difference was observed between *PTEN* antisense-delivered cells and MIS control-delivered cells (Figure 7).

### Immunohistochemical analysis of PTEN in clinical specimens

**Figure 8:**
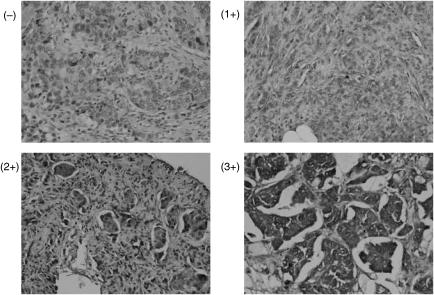
PTEN expression pattern in human breast tumours. ErbB2-overexpressing primary breast carcinomas from 17 patients who subsequently received trastuzumab plus pacliataxel were analysed. PTEN expression of these tumours was examined by IHC and semiquantified using immunoreactive scores. Representative tumour PTEN stainings (IRS 3+, 2+, 1+, and −).

**Figure 9:**
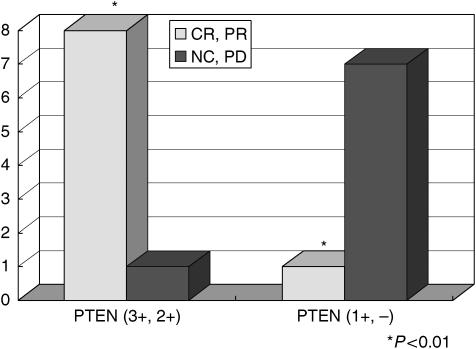
Immunohistochemical investigation of PTEN expression from patients with erbB2-overexpressing breast cancer treated with trastuzumab. We analysed 17 breast cancer tissue samples by immunohistochemistry. In those cases with increased PTEN expression (3+, 2+), the efficacy of trastuzumab-containing chemotherapy was remarkable and the response rate statistically significant (statistical analysis was performed with Fisher's test).

To explore the clinical significance of PTEN status in predicting response to trastuzumab-based therapy, we evaluated PTEN expression in 17 HER2/neu-overexpressing primary breast carcinomas from patients who subsequently developed metastatic breast cancer and received trastuzumab plus paclitaxel therapy after preliminary evaluation, and in 20 other randomly selected breast cancer patients. Immunohistochemical staining revealed that PTEN expression in these tumours was heterogeneous. Thus, PTEN expression levels were semiquantified using IRS, calculated by multiplying the percentage of PTEN-positive cells (scored 0–4) by the PTEN staining intensity (scored 1–3) (Chui *et al*, 1996). Immunoreactive scores range from 0 to 12, representing the range of PTEN staining from an undetectable level in PTEN-deficient tumours to full expression in normal individuals (Figure 8). We observed weak PTEN expression (− and 1+: IRS<7) in 40.0–49.1% of the tumours examined (eight out of 17 of the patients who received trastuzumab and paclitaxel, and eight out of 20 from the preliminary study), which is consistent with previous reports that ∼50% of breast cancers are PTEN deficient (Perren *et al*, 1999; Depowski *et al*, 2001). Of the 17 patients who received trastuzumab plus paclitaxel, those who had PTEN-deficient tumours (− and 1+: IRS<7) had significantly lower complete and partial response rates to trastuzumab therapy than those with PTEN-positive tumors (2+ and 3+ or IRS⩾7; 12.50 *vs* 88.89%, *P*=0.00337) (Figure 9).

## DISCUSSION

Several studies using either primary tumour tissue or established tumour cell lines have demonstrated a high frequency of *PTEN* mutation/deletion in various human cancers including brain, bladder, breast, prostate and endometrial cancers (Louis and Gusella, 1995; Cairns *et al*, 1997; Li *et al*, 1997; Risinger *et al*, 1997; Tashiro *et al*, 1997; Teng *et al*, 1997; Podsypanina *et al*, 1999; Mutter *et al*, 2000). *PTEN* has been found to be one of the most common targets of mutation in human cancer, with a mutation frequency approaching that of p53 (Bose *et al*, 1998). Human breast cancer is associated with a loss of heterozygosity or mutation of the *PTEN* gene, and decreased PTEN expression is associated with invasive breast cancer and poor prognosis (Singh *et al*, 1998; Garcia *et al*, 1999). The principal activity of PTEN is to dephosphorylate phosphatidylinositol 3,4,5-triphosphate (PIP3), produced by PI3K and a major activator of the cell survival kinase Akt. Thus, negative regulation of the PI3K pathway by PTEN is critical, and loss of PTEN function creates an environment conducive to tumorigenesis (Feilotter *et al*, 1999; Perren *et al*, 1999; Torres and Pulido, 2001). Currently, patients with metastatic breast cancer are selected for trastuzumab-based therapy if the primary tumour overexpresses the HER2/neu protein, or if FISH provides evidence of *HER2/neu* gene amplification (Di Cristofano *et al*, 1998). While trastuzumab therapy is not a cure for disseminated disease in *HER2/neu* amplification cases, major tumour regressions are often seen, particularly when the trastuzumab is given in combination with other chemotherapeutic agents. However, in spite of patient selection on the basis of *HER2/neu* FISH or protein overexpression, less than 30% of patients respond to trastuzumab monotherapy. Taking these observations into account, *HER2/neu* gene amplification is a necessary biomarker but not sufficient to predict the efficacy of trastuzumab. It is probable that trastuzumab efficacy is also controlled by other biomarkers associated with the downstream pathway of HER2/neu. Some recent reports have demonstrated that PTEN activation contributes to tumour inhibition by trastuzumab, and loss of PTEN predicts trastuzumab resistance *in vitro*, *in vivo* and in the clinical setting (Crowder *et al*, 2004; Nagata *et al*, 2004; Pandolfi, 2004). According to Nagata *et al* (2004), activation of PTEN in *HER2/neu*-amplified breast cancer could contribute to the efficacy of trastuzumab; it was also demonstrated that efficacy of trastuzumab in clinical samples of *HER2/neu*-amplified breast cancer is significantly associated with PTEN expression.

In this *in vitro* study, we investigated the relationship between trastuzumab's efficacy and PTEN status with a drug-resistant cell model established by continuous exposure to trastuzumab. Our method for the establishment of trastuzumab-resistant HER2/neu-overexpressing breast cancer cells is similar to that of Nahta *et al* (2004) and could provide a model for the investigation of the molecular mechanisms of trastuzumab resistance. As in previous reports (Crowder *et al*, 2004; Nagata *et al*, 2004; Pandolfi, 2004), our study revealed a remarkable difference in PTEN activity between drug-sensitive and -resistant cells. Selective inhibition of PTEN, which is strongly associated with induction of Akt activity, resulted in markedly decreased trastuzumab sensitivity. Furthermore, in the clinical samples from patients with HER2/neu-overexpressing breast cancer treated with trastuzumab, PTEN expression was strongly associated with trastuzumab efficacy. Therefore, based on this study and previous reports, trastuzumab efficacy is partly dependent on the PTEN status and degree of Akt and associated signal transduction activities in HER2/neu-overexpressing breast cancer.

Although we have not described the studies here, we have also investigated the effects of selective inhibition of other signal transduction pathways and transcription factors, including the Ras/Raf/MAPK pathway, JNK-medicated pathway and bcl2 apoptotic proteins, in trastuzumab-sensitive and -resistant SKBR3 cells. Little effect on trastuzumab efficacy was observed.

Other reports have discussed other predictive markers of trastuzumab efficacy (Albanell and Baselga 2001; Lu *et al*, 2001, 2004; Menendez *et al*, 2005; Vogel *et al*, 2005), including insulin-like growth factor-1 receptor (IGF-1R), a widely expressed protein tyrosine kinase with an important role in the suppression of apoptosis and stimulation of proliferation. However, these studies have not yet been expanded to the clinical setting (Lu *et al*, 2001). Our findings are strongly supported by clinical data and previous reports (Crowder *et al*, 2004; Nagata *et al*, 2004; Pandolfi, 2004) that PTEN-deficient tumours did not respond to trastuzumab. Therefore, PTEN, which is strongly associated with the PI3K/Akt signal transduction pathway, could predict the efficacy of trastuzumab in patients with excessive HER2/neu expression. Owing to the small cohort used in our study, further large-scale clinical investigations, including both histopathological and genetic aspects, should be carried out.

Several new molecular-target chemotherapeutic agents have been developed in recent years. Of these, selective inhibitors of Akt and mTOR have been examined in preclinical or Phase 1 clinical trials (Carraway and Hidalgo, 2004; Meuillet *et al*, 2004). According to the results of our study, the PTEN and Akt downstream pathway is critical for the efficacy of trastuzumab and these new agents targeted to the HER2/neu-mediated transduction pathway might give hope in overcoming trastuzumab resistance.
